# Efficacy of Expansion Pharyngoplasty without Drug-induced Sleep Endoscopy Screening in Obstructive Sleep Apnea

**DOI:** 10.1055/s-0044-1782630

**Published:** 2024-10-25

**Authors:** Rafael Tenor, Juan Miguel Palomeque-Vera, Angel Bandera-López, Pilar Cuellar, Manuel Oliva-Domínguez

**Affiliations:** 1 Department of Otolaryngology, Head and Neck Surgery, Hospital Universitario Costa del Sol, Marbella, Spain; 2 Department of Radiology and Physical Medicine, Ophthalmology and Otorhinolaryngology, Faculty of Medicine, University of Málaga, Spain; 3 Department of Pneumology, Hospital Universitario Costa del Sol, Marbella, Spain

**Keywords:** sleep apnea, surgical procedures, endoscopy

## Abstract

**Introduction** Expansion sphincter pharyngoplasty has been shown to be a good alternative to continuous pressure devices in patients with moderate to severe obstructive sleep apnea. On the other hand, drug-induced sleep endoscopy provides information on the pattern of collapse in obstructive sleep apnea, although it is unclear whether this information improves the surgical outcomes.

**Objective** To evaluate the success rate obtained when performing expansion sphincter pharyngoplasty on a group of patients diagnosed with moderate to severe obstructive sleep apnea who were not previously selected by drug-induced sleep endoscopy.

**Methods** We present a series of patients with moderate to severe obstructive sleep apnea who underwent surgery. Pre- and postoperative home sleep apnea tests were performed. The success rate was calculated, and we assessed whether there were statistically significant pre- and postoperative differences in the apnea-hypopnea index and oximetry values.

**Results** In total, 20 patients were included, and the surgical success rate was of 80%. Statistically significant improvements were demonstrated in the mean apnea-hypopnea index (from 40.25 ± 15.18 events/hour to 13.14 ± 13.82 events/hour; *p* < 0. 0001), the mean oximetric data (from 26.3 ± 12.97 desaturations/hour to 13.57 ± 15.02 desaturations/hour; *p* = 0.034), and in the mean percentage of total sleep time in which the patient had less than 90% of saturation (from 8.64 ± 9.25% to 4.4 ± 7.76%; *p* = 0.028).

**Conclusion** The results showed significant improvements in the apnea-hypopnea index and in the oximetric data, with a surgical success rate of 80%, despite the lack of prior drug-induced sleep endoscopy screening.

## Introduction

Obstructive sleep apnea (OSA) can be considered a first-magnitude public health problem. It is estimated that around half of the world population suffers from this disorder, 1 which is associated with significant morbidity and mortality.

Nowadays, continuous positive airway pressure (CPAP) devices are recommended in cases of moderate to severe OSA which presents with associated symptoms or hypertension, as well as hygienic-dietary measures. 2 Although the efficacy of these devices has been widely demonstrated, the low adherence on the part of the patients represents a limitation. Upper airway (UA) surgery is considered an alternative treatment; it has proved to be effective in some patients, but it is not widely used, as its outcomes cannot be predicted.

The field of UA surgery in OSA has undergone great changes in the past 20 years. Surgical techniques have evolved from resective surgeries of the palate to reconstructive surgeries, in which attempts are made to preserve the mucosa, soft tissues, and respect the musculature and function of each structure. The collapse of the lateral walls of the pharynx seems to contribute decisively to the obstructive phenomenon, especially in the most severe degrees of OSA, in which the multilevel collapse pattern is more frequent. People with OSA present pharyngeal sidewalls that are thicker and more prone to collapse than those without OSA. 3 This is known, among other things, thanks to drug-induced sleep endoscopy (DISE), a procedure that enables the individual assessment of the collapse pattern, as well as of the level or levels of the UA affected and to what degree. Popularized as a method to perform topodiagnosis of the problem, in theory DISE would help to optimize the treatment through the selection the ideal surgical technique. Expansion sphincter pharyngoplasty (ESP), described in 2007 4 (with some subsequent modifications), tries to correct the lateral collapse by sectioning and transposing the palatopharyngeal muscle. Easy to perform and with little associated morbidity, it is a therapeutic alternative in those people affected by moderate to severe OSA and in those in whom lateral collapse is suspected.

## Study Objectives

To assess the results of ESP in a group of patients with moderate to severe OSA, without prior selection by DISE but selected by examination in the office. These patients did not tolerate or refused the use of CPAP and underwent ESP in our center from 2019 to 2022.

## Methods

The present is a series of cases diagnosed with moderate to severe OSA by type-3 home sleep apnea test (HSAT), who underwent ESP, according to the Pang technique modified by Sorrenti, 5 in the Otolaryngology Department of Costa del Sol university hospital between 2019 and 2022.

The present study was approved by the Ethics in Research Committee of our hospital.

### Selection Criteria

The inclusion criteria were age > 18 years, diagnosis of moderate to severe OSA (apnea-hypopnea index [AHI] > 15 events/hour), and intolerance or rejection of CPAP. The exclusion criteria were body mass index (BMI) ≥ 33 Kg/m ^2^ , poor nasal ventilation (in this case, previous nasal surgery was proposed), significant craniofacial anomalies, serious comorbidities, or score > 3 on the American Society of Anesthesiologists (ASA) classification.

### Study Variables

We collected sociodemographic and clinical data, including age at the time of the surgery, sex, weight, height, BMI, AHI, the oxygen desaturation index (ODI), and the percentage of the total sleep time in which the patient had a saturation lower than 90% (T90%). Examinations by an ear, nose, and throat (ENT) surgeon included nasoscopy, fibroscopy of the UAs and staging of tonsil size (scale from 0 to 4), as well as tongue position (scale from 1 to 4), leading to the Friedman staging system: stage I – large tonsils and low tongue position; stage II – large tonsils and high tongue position, or small tonsils and low tongue position; and stage III – small tonsils and high tongue position. 6

Patients underwent major outpatient surgery, with a hospital stay of fewer than 24 hours in all cases. Three different surgeons performed the interventions. Previous DISE was not performed, and the level or levels of collapse were estimated according to the findings of the fibroscopic exploration in the office, with the help of the Müller maneuver and simulated snoring. In some cases, nasal repermeabilization surgery was previously performed. After 3 to 12 months of the surgery, a new HSAT was performed.

### Surgical Success and Statistical Method

Surgical success was considered in those patients who presented at least a 50% reduction in AHI, and this was below 20 events/hour. We also calculated a more restrictive success rate, when the reduction achieved was of at least 50% of the pre-surgical AHI and the postoperative AHI was < 15, and the cure rate, when AHI was < 5. The quantitative variables are presented as mean and standard deviation values, and the qualitative variables, as percentages and number of cases. We also calculated whether there were significant differences in the pre- and postoperative values of the AHI, ODI and T90% with the Wilcoxon test. Values of *p* ≤ 0.05 were considered statistically significant.

### Surgical Procedure

The procedure is performed under general anesthesia, with the patient in the supine position and under orotracheal intubation, with a Boyle-Davis mouth gag within the oral cavity. A bilateral tonsillectomy is performed. The palatopharyngeus muscle is identified; its inferior end is transected horizontally with electrocautery and rotated superolaterally. The muscle is isolated and left with its superoposterior surface attached to the pharyngeal constrictor muscles. Sufficient muscle must be isolated to mobilize the muscle and to enable its suturing with Vicryl (Ethicon, Inc., Bridgewater, NJ, United States) to the hamulus area in position to obtain an anterolateral palate stretching. Then, instead of performing an incision on the anterior pillar arch, a tunnel is created from the apex of the tonsillar fossa to the hamulus, placing the muscle flap in the tunnel and anchoring it with a stich in the hamulus area. A partial uvulectomy is performed. The anterior and posterior tonsillar pillars are then apposed with Vicryl sutures.

## Results

### Patient Characteristics

In total, 20 patients were included, and 95% (19) were men and 5% (1) were women. The mean age on the day of surgery was of 45.7 ± 10.41 years, and the mean BMI was of 28.22 ± 2.94 Kg/m ^2^ . As for the Friedman stage, 50% (10) of patients were in stage III, 40% (8), in stage II, and 10% (2), in stage I.

### Findings

**Fig. 1:**
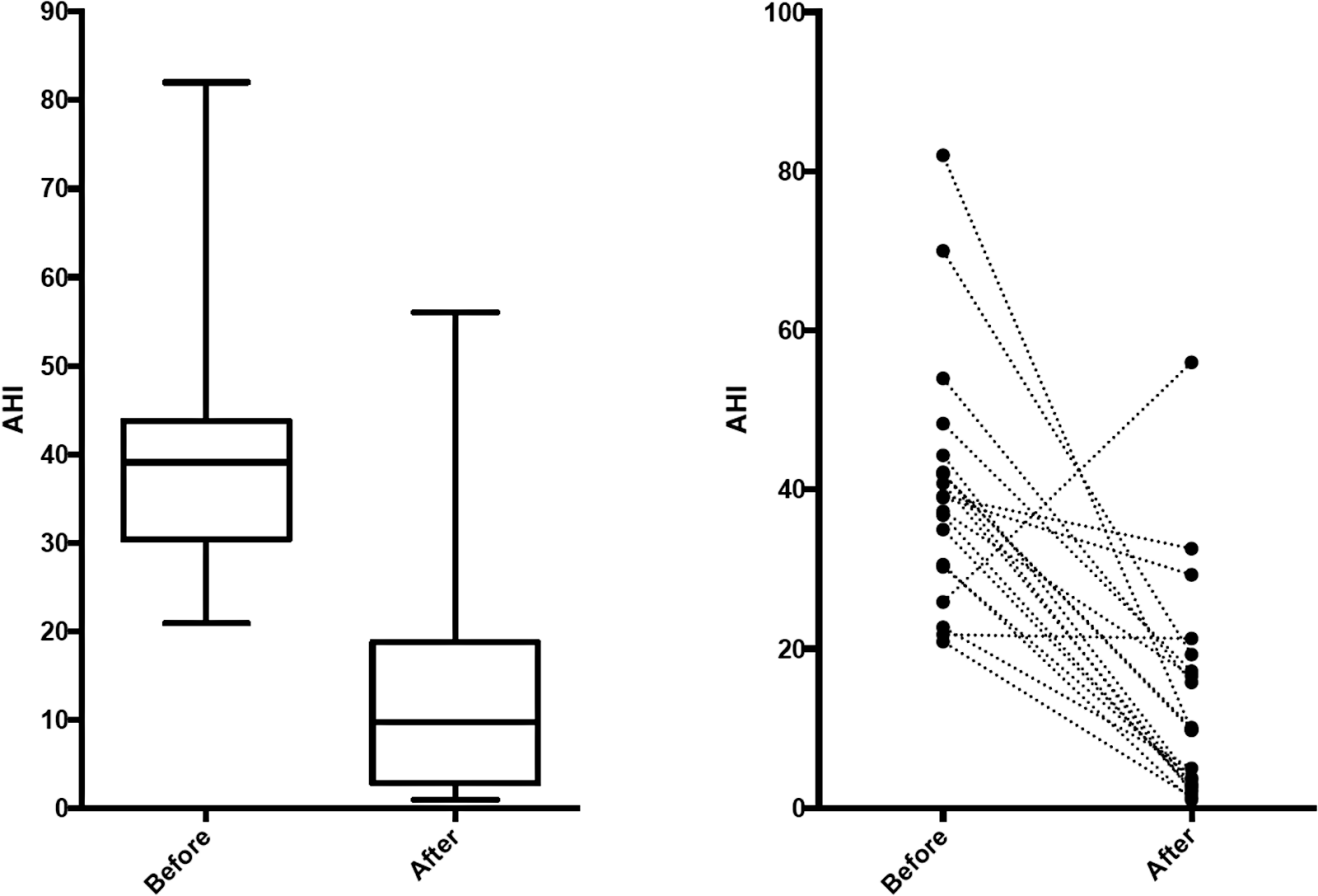
Boxplots and lines showing the apnea-hypopnea index (AHI) on two different sleep tests (before and after surgery).

The mean preoperative AHI was of 40.25 ± 15.18 events/hour, and the mean postoperative AHI was of 13.14 ± 13.82 events/hour. A significant difference was demonstrated in the AHI before and after surgery ( *p* < 0.0001). ( Fig. 1 )

**Fig. 2:**
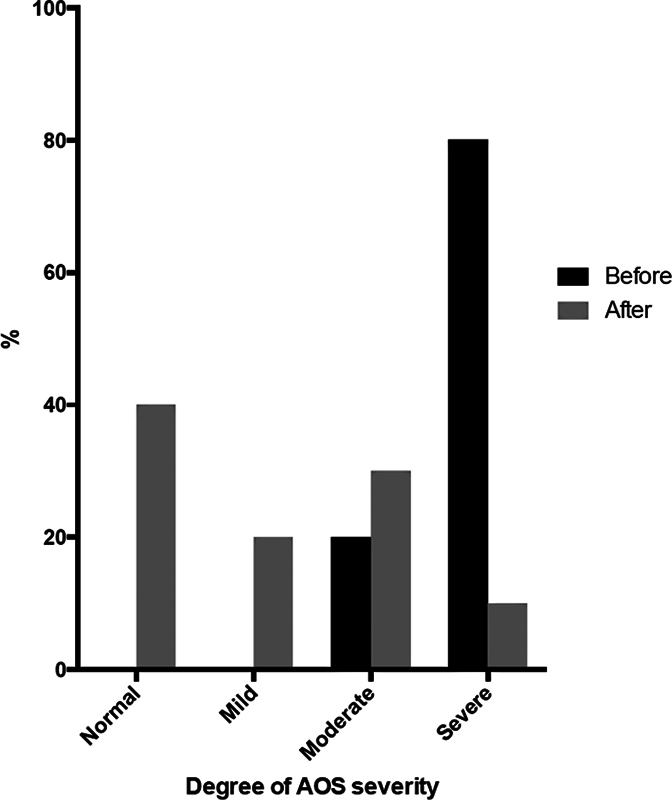
Classification of patients according to the degree of severity of obstructive sleep apnea (OSA) before and after surgery.

Preoperatively, 80% (16) of the patients had severe OSA and 20% (4), moderate OSA. Postoperatively, 40% (8) did not present OSA, 20% (4) presented mild OSA, 30% (6) moderate OSA, and 10% (2), severe OSA. ( Fig. 2 )

The surgical success rate was of 80% (16). The most restrictive success rate (AHI decrease of at least 50% and below 15 events/hour) was of 60% (12). Cure was achieved in 40% (8) of the patients. In all cases except one, the AHI improved.

Regarding the oximetric data, the mean ODI significantly decreased after surgery (26.3 ± 12.97 desaturations/hour versus 13.57 ± 15.02 desaturations/hour; *p* = 0.034). The mean T90% also presented a significant improvement after surgery (8.64 ± 9.25% versus 4.4 ± 7.76%; *p* = 0.028).

## Discussion

The results obtained in the present series of patients are like those reported previously ( Table 1 ). However, the patients did not undergo DISE prior to surgery, and we highlight that in the current case series 80% of the participants had severe OSA. There was an improvement in both in the AHI and in the nocturnal oximetric data. Although the percentage of “cured” cases (AHI ≤ 5 events/hour in 40% of cases) in the present study is not insignificant, we believe, as other authors 7 have already expressed, that the goal of surgery is not to cure a condition which is obviously incurable, but to control the symptoms and minimize ongoing multisystem damage. We consider surgery only for those patients who have failed CPAP, as a salvage treatment, because a partial improvement of the problem is preferable to an untreated patient. Traditionally, OSA surgery has been criticized as ineffective, 8 especially considering the results of surgical techniques now in disuse, such as uvulopalatopharyngoplasty (UPPP), whose results seem to be improved by ESP, the heterogeneity of surgical techniques used, and the idea that a successful case should also be a cured case, with an AHI > 5.

**Table 1:** Surgical success rate in different series from the literature of patients undergoing ESP

Author	Carrasco-Llatas et al. 9	Pang et al. 10	Güler et al. 11	Bosco et al. 12	Lorusso et al. 13	Hong et al. 3	Cayir et al. 14	Ciğer et al. 15
Year	2015	2016	2018	2019	2018	2019	2021	2022
N	10	73 a, b	67 b	17 b	20 c	63 a	39	91 a, b
Mean age (years)	38.6 ± 6.3	46.8 (25–67)	NA	42.06 (27–72)	50 ± 8	42.1 (20–54)	43.2 ± 7.5	NA
Mean BMI (Kg/m ^2^ )	26.3 ± 3.2	25.5 (20.3–31.2)	27.4 ± 2.7	27.97 ± 2.4	28.6 ± 3.1	27.6 (19–32.1)	NA	30.3 ± 3.8
Mean preoperative AHI (events/hour)	27.7 ± 7.5	26.3 ± 17.7	18.3 ± 2.2	29.78 ± 15.99	41.7 ± 21.5	35.5 ± 10.7	25.2 ± 8.3	33.4 ± 13.6
Mean postoperative AHI (events/hour)	6.5 ± 5.2	12.6 ± 5.8	8 ± 1	11.51 ± 7.76	17.4 ± 8.9	17.3 ± 8.9	11.6 ± 6.9	NA
Success rate (%)	90	86.3	67.2	82.35	65	67	72	83.5
DISE	Yes	Some	No	Yes	No	Yes	No	Yes

**Abbreviations:** AHI, apnea-hypopnea index; BMI, body mass index; DISE, drug-induced sleep endoscopy; ESP, expansion sphincter pharyngoplasty; NA, not available.

**Notes:**^a^ ESP is combined with anterior palatoplasty; ^b^ includes patients with mild OSA; ^c^ some undergoing multilevel surgery.

Obtaining the expansion and stabilization of the pharyngeal airspace is the key of the modern OSA surgeries. Unlike previous approaches like UPPP, in which the surgical goal is to shorten and stiffen the soft palate, the main purpose of the new techniques is the stiffening and enlargement of the lateral walls. Lateral pharyngoplasty and, later, ESP have shown better results in comparison with UPPP. Different variations of the ESP procedure have been described, such as that of Sorrenti 5 (functional expansion pharyngoplasty) or Lorusso 13 16 (modified expansion pharyngoplasty). Both modifications avoid scarring of the velum and aim to achieve an even less aggressive and more “physiological” approach to the lateral pharynx wall and soft palate.

The best tool to assess the pattern of collapse of the UA in OSA seems to be DISE. Its development and popularization have helped us to better understand the mechanics of the UA in OSA, and it has been proposed as an interesting tool to optimize the preoperative selection of patients. 17 Based on this acquired knowledge, we know that the most severe cases of OSA usually correspond to a multilevel collapse pattern, in which the soft palate is almost always involved. 18 Therefore, considering the time and resources that DISE requires (such as previous training to be able to interpret what is observed, the need to have an anesthetist who also knows the procedure, and the lack of resources available), we do not perform DISE by default on all patients. Despite everything, the results obtained do not seem to be bad, something that is also reflected in the literature: there is no solid evidence that previous screening with DISE improves the results of OSA surgery. 19 20 21 Although previous screening with DISE seems to help in treatment planning, its impact has not been demonstrated, given the heterogeneity of the published data and the poor quality of the existing publications. In addition, an incorrectly-interpreted DISE can lead to erroneous surgical planning, a drag on the outcome. It is worth noting a prospective and randomized clinical trial published in 2022 22 in which an improvement in the results was found in the group of OSA patients screened with DISE who underwent surgery of the soft palate (barbed reposition pharyngoplasty).

### Study Limitations

Among the limitations of the present study, we can mention the small number of patients (strongly impacted by the COVID-19 pandemic) and not having calculated the postoperative BMI due to lack of weight data for many patients. Besides, no data were collected on pre- and postoperative quality of life and sleepiness, interesting metrics to know to get a complete picture of the impact of surgery on these patients.

## Conclusion

The ESP procedure without previous DISE appears to be an effective therapeutic alternative to CPAP in patients with moderate to severe OSA. The results obtained in the present study showed a significant improvement in the AHI and oximetric data, with a success rate of 80%.

In the absence of DISE, we suggest that the Muller maneuver with an accurate clinical evaluation can be considered a useful tool for the selection of patients to undergo surgery.

Well-designed studies are needed to validate the use of ESP as a treatment for moderate to severe OSA and to assess the cost-effectiveness of DISE as a preoperative screening tool.
